# Design of Substrate-Integrated Waveguide Loading Multiple Complementary Open Resonant Rings (CSRRs) for Dielectric Constant Measurement

**DOI:** 10.3390/s20030857

**Published:** 2020-02-06

**Authors:** Honggang Hao, Dexu Wang, Zhu Wang

**Affiliations:** College of Electronic Engineering, Chongqing University of Posts and Telecommunications, Chongqing 400065, China; S180401004@stu.cqupt.edu.cn (D.W.); S180431033@stu.cqupt.edu.cn (Z.W.)

**Keywords:** dielectric constant, cavity, substrate-integrated waveguide, complementary open resonant ring, sensor

## Abstract

In order to solve the low-sensitivity problem of the dielectric constant with the resonant cavity method, a sensor based on a substrate-integrated waveguide structure loaded with a multi-complementary open resonant ring is proposed. With the enhanced resonance characteristics of the sensor, this design realized the measurement of complex dielectric constants in a wide range. The frequency selectivity of the sensor is improved by the high-quality factor of the substrate-integrated waveguide. By loading three complementary resonant rings with different opening directions in the ground plane, a deeper notch and better out-of-band suppression are achieved. The effect of the complex dielectric constant on both resonant frequency and quality factor is discussed by calculating the material under test with a known dielectric constant. Simulation and experimental results show that a resonance frequency offset of 102 MHz for the per unit dielectric constant is achieved. A wide frequency offset is the prerequisite for accurate measurement. The measurement results of four plates match well with the standard values, with a relative error of the real part of the dielectric constant of less than 2% and an error of less than 0.0099 for the imaginary part.

## 1. Introduction

The dielectric constant of a substance is an important characteristic parameter that characterizes the electromagnetic properties of a material. Accurate measurement of the dielectric constant is particularly important in the development, production, and use of microwave dielectric materials [1,2,3,4,5,6]. Traditional methods for measuring the dielectric constant mainly include the transmission line method, waveguide method, free space method, neural network method, and resonant cavity method [7,8,9,10,11,12,13]. In the resonant perturbation methods, the material under test (MUT) is placed in a resonant cavity. The resonance frequency and quality factor (*Q*) are affected by the permittivity of the MUT [14]. Then, the permittivity of the MUT can be obtained by measuring the resonant frequency shift and the *Q* variation of the cavity [15,16]. This method is simple, and it is suitable for measurements of small sizes, with a low dielectric constant value and low-loss material [17,18]. A sensor applied to the measurement of complex dielectric constants of solid substances was designed in the literature by using resonant cavity perturbation technology [19], which is based on a substrate-integrated waveguide (SIW) structure. The measured frequency range was 7.075–7.2 GHz, and the measurement error of the dielectric constant did not exceed 6%. A novel planar symmetric splitter ring resonating cavity (SSRR) sensor structure was proposed in the literature [20]. The sensor was used to characterize the dielectric properties of common solid materials. The dielectric constants of three common plates were measured, with a frequency detection range of 2.06–2.2 GHz and a relative error of less than 1.3%.

Although the resonant cavity method has a high accuracy, the measurement frequency only works for a point frequency or narrow bandwidth measurement. The resonance characteristics will become poor (|S_11_| ≥ −10 dB) as the measured frequency widens, due to impedance matching. Inversion of the dielectric constant from a small frequency offset cannot achieve a high-sensitivity measurement. In order to solve these problems, a sensor for measuring complex dielectric constants in a wide range is realized by loading multiple complementary open resonant rings (CSRRs). The SIW integrates the traditional waveguide structure into the substrate to achieve miniaturization of the resonant cavity. Three CSRR loops with different opening directions realize a deeper notch at the resonant frequency point and enhance the out-of-band suppression of the sensor, to achieve a wider frequency offset for the change in dielectric constant. In this way, sensitivity is improved.

## 2. Theoretical Analysis

The complex dielectric constant of an object can be divided into real and imaginary components such as *ε* = *ε*′ − *jε*″. The tangent of the dielectric loss angle, tan*δ*, represents the relationship between the imaginary part and the real part of the dielectric constant of an object [21]:

(1) $$\tan\delta =\frac{\varepsilon^{\prime \prime }}{\varepsilon^{\prime }}$$

According to the principle of measuring the complex dielectric constant by the resonant cavity method, the MUT is placed in a resonant cavity. The resonance frequency and *Q* are affected by the permittivity of the MUT. Then, the permittivity of the MUT can be obtained by measuring the resonant frequency shift and the *Q* variation of the cavity according to the following relationships [22,23]:

(2) $$\varepsilon_{s}^{\prime }=A\cdot \varepsilon_{c}^{\prime }\frac{V_{c}}{V_{s}}(\frac{f_{c}-f_{s}}{f_{c}})+\varepsilon_{c}^{\prime }$$

(3) $$\varepsilon_{s}^{\prime \prime }=B\cdot (\frac{\varepsilon_{c}^{\prime }{}^{2}+\varepsilon_{c}^{\prime \prime }{}^{2}}{\varepsilon_{c}^{\prime }})\frac{V_{c}}{V_{s}}(\frac{Q_{c}-Q_{s}}{Q_{c}Q_{s}})+\frac{\varepsilon_{s}^{\prime }\varepsilon_{c}^{\prime \prime }}{\varepsilon_{c}^{\prime }}$$

where $\varepsilon_{s}^{\prime }$ and $\varepsilon_{c}^{\prime }$ are the real parts of the relative dielectric constant of the MUT and the dielectric substrate of the resonant cavity, respectively, and the corresponding imaginary parts are $\varepsilon_{s}^{\prime \prime }$ and $\varepsilon_{c}^{\prime \prime }$. *V_c_* and vs. are the volumes of the resonator and the MUT, respectively. *f_c_* and *f_s_* represent the resonant frequency of the empty cavity and that of the filled cavity, respectively. The quality factors of the resonant cavity before and after perturbation with the MUT are *Q_c_* and *Q_s_*, respectively. *A* and *B* are constants. When the physical dimensions of the resonant cavity and the MUT are determined, $A\varepsilon_{c}^{\prime }\frac{V_{c}}{V_{s}}$ and $B(\frac{\varepsilon_{c}^{\prime }{}^{2}+\varepsilon_{c}^{\prime \prime }{}^{2}}{\varepsilon_{c}^{\prime }})\frac{V_{c}}{V_{s}}$ are constant, and Equations (2) and (3) can then be simplified into

(4) $$\varepsilon_{s}^{\prime }=A_{1}\cdot \Delta f+B_{1}$$

(5) $$\varepsilon_{s}^{\prime \prime }=C_{1}\cdot \Delta Q+D_{1}$$

where

(6) $$\Delta f=\frac{f_{c}-f_{s}}{f_{c}}$$

(7) $$\Delta Q=\frac{Q_{c}-Q_{s}}{Q_{c}Q_{s}}$$

Equations (4) and (5) are fitted by using standard media with a known dielectric constant. The results of the fitting can be used to measure the complex dielectric constant of the MUT.

## 3. Sensor Design

**Figure 1:**
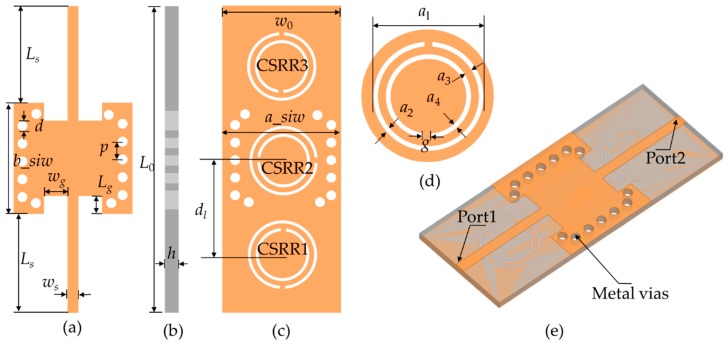
Multi-CSRR model structure. (**a**) Top view; (**b**) lateral view; (**c**) bottom view; (**d**) the structure of CSRR; (**e**) 3-D view. Dimensions are: *W*_0_ = 12.0 mm, *L*_0_ = 21.2 mm, *h* = 0.787 mm, *d* = 1.0 mm, *p* = 1.8 mm, *W*_s_ = 1.1 mm, *L_s_* = 10.0 mm, *L_g_* = 1.8 mm, *W_g_* = 2.4 mm, *g* = 0.5 mm, *a*_1_ = 7.0 mm, *a*_2_ = 0.3 mm, *a*_3_ = 0.5 mm, *a*_4_ = 0.3 mm, *d_l_* = 9.0 mm.

The sensor based on the SIW-CSRR is shown in Figure 1. A Rogers 5880 plate with a relative permittivity of 2.2 and dielectric loss of 0.0009 is selected as the substrate. The sensor is fed by 50 Ω microstrip line matching, and port 1 and port 2 are directly fed by two 50 Ω SMA connectors. Three CSRR loops with different opening directions are etched on the under layer of the substrate.

**Figure 2:**
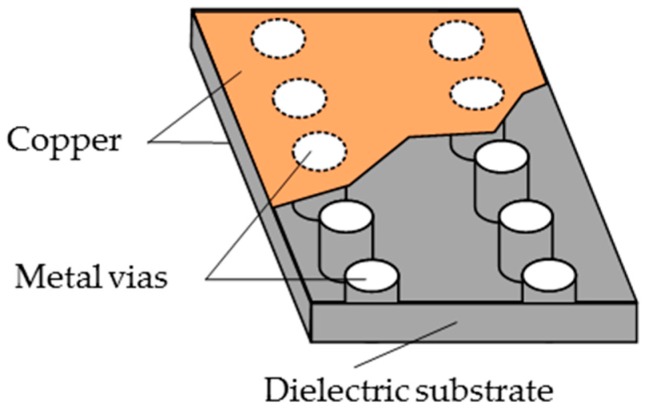
SIW model structure.

The SIW is a kind of new wave guide structure, which is integrated on a dielectric substrate with low insertion loss and low radiation features. The top and bottom surfaces of the dielectric substrate are metal layers, and the vias of the SIW are metal. The SIW combines the advantages of the microstrip line and metal rectangular waveguide, which has a small size, low insertion loss, and low radiation features. Its structure is shown in Figure 2. The resonant frequency of the SIW is determined by the effective width and length, as shown in Equation (8):

(8) $$f_{mn0}=\frac{1}{2\sqrt{\mu \varepsilon }}\sqrt{{\left(\frac{m}{a\_eff}\right)}^{2}+{\left(\frac{n}{b\_eff}\right)}^{2}}$$

where *m* and *n* are the mode indices, the permittivity and permeability are *ε* and *μ*, respectively, and *a_eff* and *b_eff* represent the SIW’s effective width and length, respectively. They can be approximated according to the following relationships [24]:

(9) $$a\_eff=a\_siw-\frac{d^{2}}{0.95p}$$

(10) $$b\_eff=b\_siw-\frac{d^{2}}{0.95p}$$

where *d* and *p* are the diameter of the vias and the spacing between the vias, respectively. *a_siw* and *b_siw* are the width and length of the resonant SIW cavity, respectively. The SIW cavity is excited by a 50 Ω microstrip transmission line with a line width of 1.15 mm. The inset feeding method is used for impedance matching.

**Figure 3:**
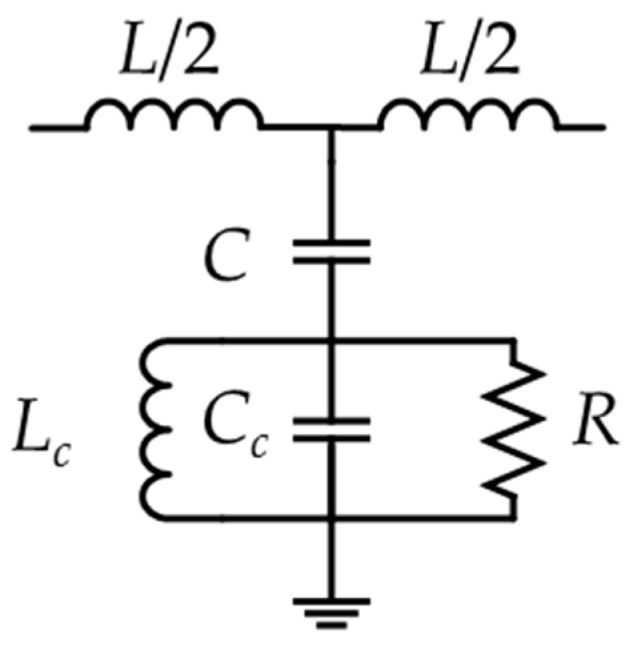
Equivalent circuit of complementary open resonant ring (CSRR).

The CSRR is a dual structure of an open resonant ring (SRR), known as a complementary open resonant ring, which is realized by etching gaps in the shape of an open resonant ring in the metal plane [25]. Its structure is shown in Figure 1d, and the equivalent circuit can be seen in Figure 3, where *L_c_* and *C_c_* are the equivalent inductance and capacitance of the CSRR. *L* is the equivalent inductance of the microstrip transmission line, *R* is the resistance of the CSRR, and *C* is the coupling capacitance between the CSRR and the microstrip transmission line. Those parameters can be extracted by an LC circuit with a model of a bandpass filter [26,27,28]. The calculated lumped parameters are presented in Table 1.

The resonant frequency of the CSRR is determined by

(11) $$f_{csrr}=\frac{1}{2\pi \sqrt{L_{c}C_{c}}}$$

The CSRR is mainly stimulated by an electric field, which is perpendicular to the ring plane and the magnetic field, which is parallel to the ring plane [29]. However, the efficiency of the magnetic field is less than that of the electric field; thus, the CSRR is mainly excited by electric fields [1]. The CSRR can be equivalent to an electric dipole when it works near the resonant frequency. A negative dielectric constant will be generated near the resonant frequency, which has a band resistance effect; because of this, there will be a resonance absorption peak [30].

**Figure 4:**
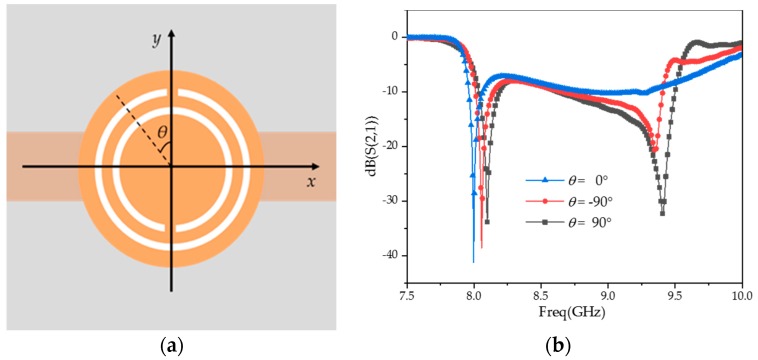
The cross-polarization characteristics of the CSRR. (**a**) CSRR–microstrip transmission line; (**b**) simulated transmission coefficients under various *θ*.

Different opening directions of the CSRR have different electromagnetic cross-coupling resonances because of the cross-polarization characteristics [31,32,33]. As shown in Figure 4a, the angle between the opening direction of the CSRR and the y direction is *θ*. The transmission coefficients, S_21_, are given in Figure 4b at *θ* = −90°, 0°, and 90°.

As can be seen from Figure 4b, there are two stopbands. The first one is independent of *θ* and corresponds to the electric polarization resonance. The second stopband is related to *θ* and corresponds to the cross-polarized resonance. When *θ* = 0°, the CSRR is symmetric about the direction of the magnetic field and there is no cross-polarization. When *θ* becomes larger, the asymmetry of the CSRR about the magnetic field direction increases, the cross-polarization effect increases, and the stopband deepens. In this work, the opening directions of the rings on the left and right sides are parallel to the microstrip line, while the opening directions of the middle of three loops are perpendicular to the microstrip line. The three CSRR loops have the same size and electric polarization resonance, which can enhance the stopband effect of the frequency band. This structure can realize not only a deeper notch but also a better out-of-band suppression. Their main advantage is the improvement in sensor sensitivity.

**Figure 5:**
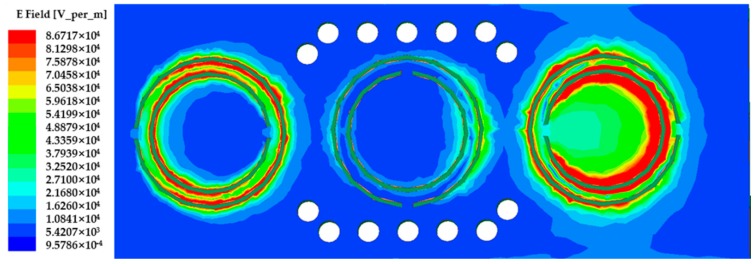
The electric field distribution of the sensor.

In order to find the best position to place the MUT, the electric field distribution of the sensor is shown in Figure 5. As can be seen from Figure 5, on the ground plane, the electric field gathers around the gap of the CSRR, and the intensity reaches 8.67 × 10^4^ V/m.

**Figure 6:**
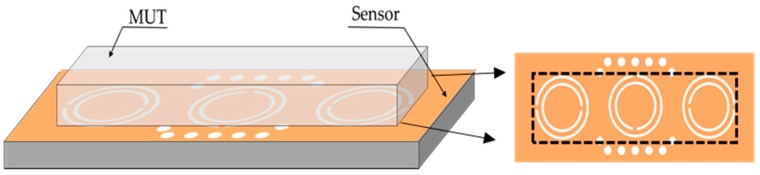
Perspective view of material under test (MUT) placed over ground plane.

The MUT is placed on top of the CSRR, where the electric field distribution is at its maximum; thus, the sensitivity is increased. The horizontal dimension of the MUT is 27.0 × 4.0 mm in order to cover the three CSRR loops. The proposed sensor is then loaded with the MUT, as shown in Figure 6.

**Figure 7:**
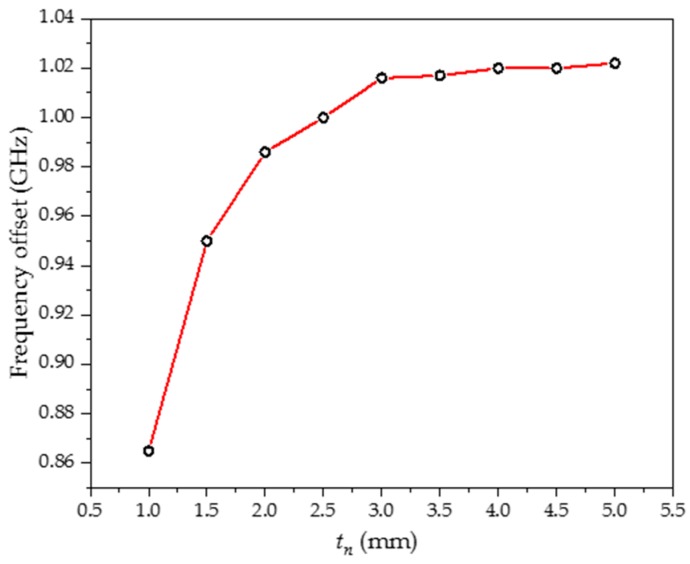
Frequency offset for different thicknesses of the MUT.

Figure 7 shows the frequency offset at different thicknesses (*t_n_*) of the MUT as the permittivity changes from 1 to 10. It may be observed that the frequency offset is almost constant for *t_n_* greater than 3 mm. Therefore, the size of the MUT is determined as 27.0 × 4.0 × 3.0 mm.

**Figure 8:**
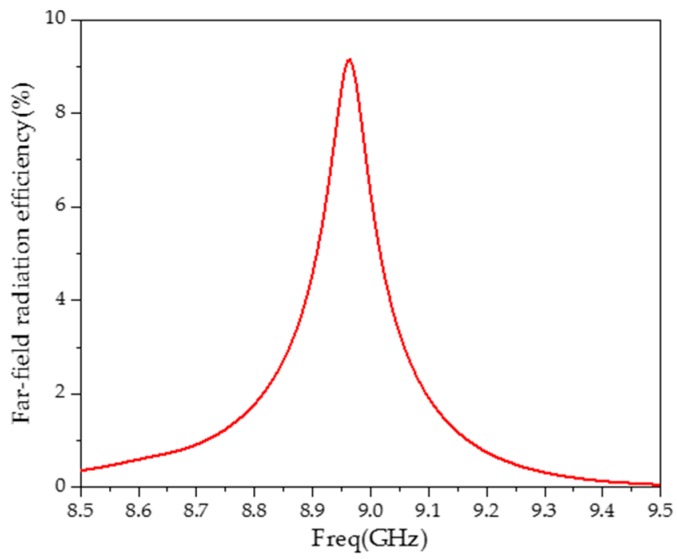
Far-field radiation efficiency of the sensor.

As the structure is not closed, the far-field radiation capability of the sensor needs to be considered as low as possible. When the radiation efficiency of the far-field is low, the measured resonant frequency and quality factor can be regarded as derived from the properties of the MUT. Figure 8 is the far-field radiation efficiency of the sensor without the MUT.

As shown in Figure 8, at the resonant frequency, the sensor has the highest far-field radiation efficiency (9.1%). On both sides of the resonant frequency, the far-field radiation efficiency sharply drops. It can be seen that the far-field radiation has little influence on the test results and the sensor has strong anti-interference properties.

**Figure 9:**
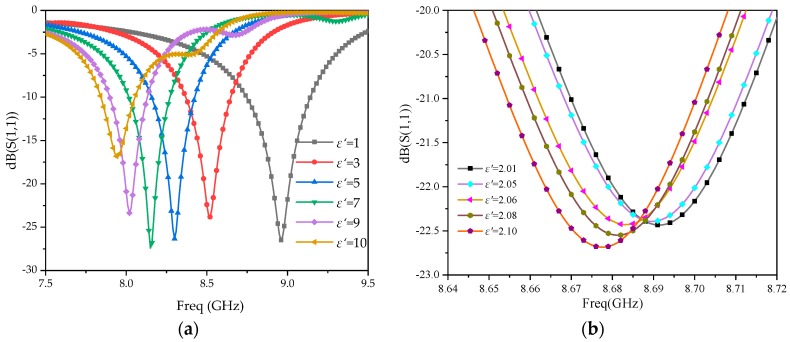
Simulated reflection coefficients under various dielectric constants of MUTs. (**a**) *ε*′ = 1–10; (**b**) *ε*′ = 2.01–2.10.

The HFSS is used to simulate the S_11_, corresponding to each MUT, which is also plotted with the dielectric constant variation, as shown in Figure 9, where the dielectric constant varies in a wide range from 1 to 10.

As can be seen in Figure 9a, when the dielectric constant of the MUT changes from 1 to 10, the resonant frequency changes from 8.961 to 7.945 GHz, achieving a wide range of measuring frequencies. Figure 9b is the S_11_ with the frequency changing from 8.64 to 8.72 GHz. It can be seen in the local enlarged figure (Figure 9b) that when 0.001 GHz of the measurement step frequency is achieved, a higher identification accuracy of 0.01 can be recognized for the dielectric constant.

**Figure 10:**
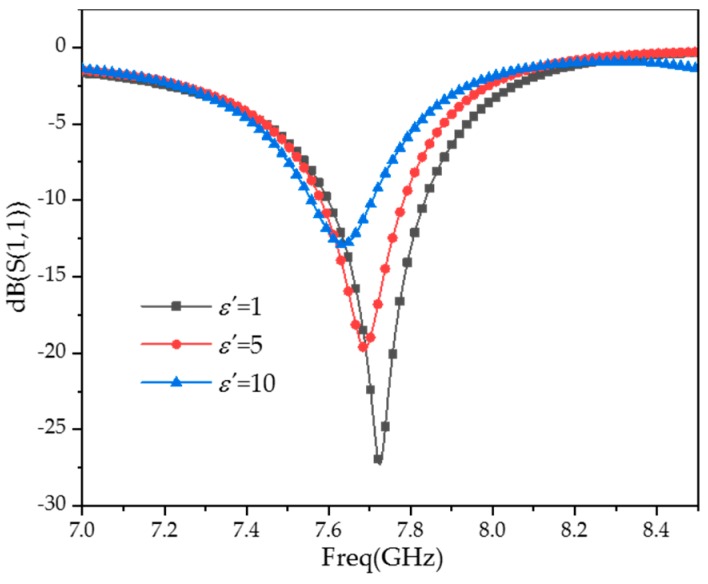
The frequency offset of different permittivities with one CSRR added.

The frequency offset of different permittivities with one CSRR added has been simulated. The results are shown in Figure 10, which show that when the permittivity of the MUT changes from 1 to 10, the frequency offset is only 91 MHz, which is less sensitive than the three CSRR loops loaded (1016 MHz). It further validates that loading multi-CSRR loops can improve the sensitivity of the sensor.

**Figure 11:**
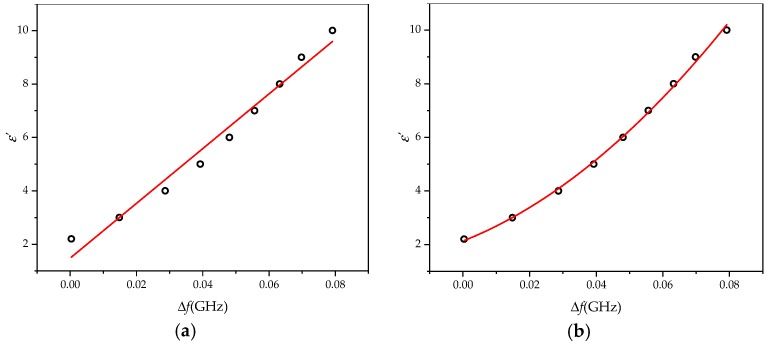
Fitting curves. (**a**) Linear fitting; (**b**) polynomial fitting.

Reading under the different relative dielectric constants in Figure 9a, the resonant frequency of *f_s_* and Equation (6) are used to calculate Δ*f*, fitting the *ε*′–Δ*f* relationship shown in Figure 11a.

Figure 10 shows the fitting curves of *ε*′ and Δ*f*. It can be seen that *ε*′ and Δ*f* basically match the linear relationship of Equation (4), while the fitting degree is 0.97613. The relative error is large, with a maximum of 31%. Quadratic fitting was adopted to correct the error of the data fitting. Then, the *ε*′–Δ*f* relations are shown in Figure 11b; the fitting degree is 0.99812, the relative error is less than 2%, and the accuracy of the dielectric constant measurement has been improved. At this point, Equation (4) is amended as

(12) $$\varepsilon_{s}^{\prime }=666.05\Delta f^{2}+49.203\Delta f+2.1296$$

When tanδ ≠ 0, there is loss of the material, and the imaginary part of the dielectric constant will affect the quality factor of the resonator. The group delay method to solve the quality factor *Q* of the resonant cavity is based on the following equation [34,35]:

(13) $$Q=\frac{w_{0}\left|\tau_{S_{11}}(w_{0})\right|}{4}=\frac{\pi f_{0}\left|\tau_{S_{11}}(f_{0})\right|}{2}$$

where *f*_0_ represents the resonance frequency and $\tau S_{11}(f_{0})$ is the group delay under the resonance frequency.

**Figure 12:**
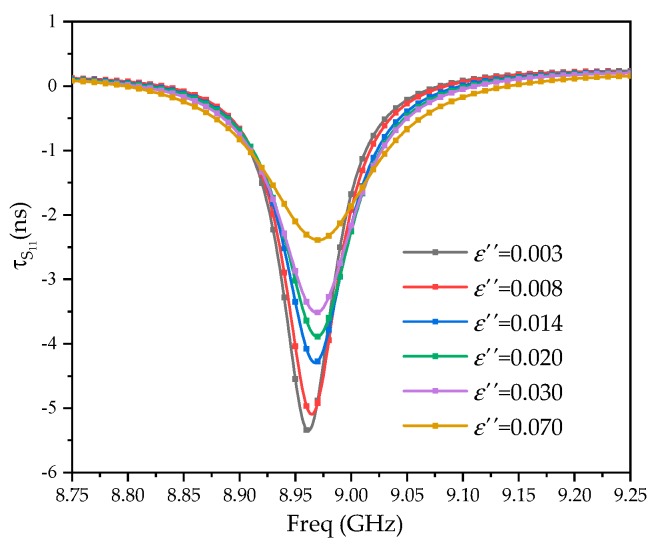
The group delay under various dielectric constants.

**Figure 13:**
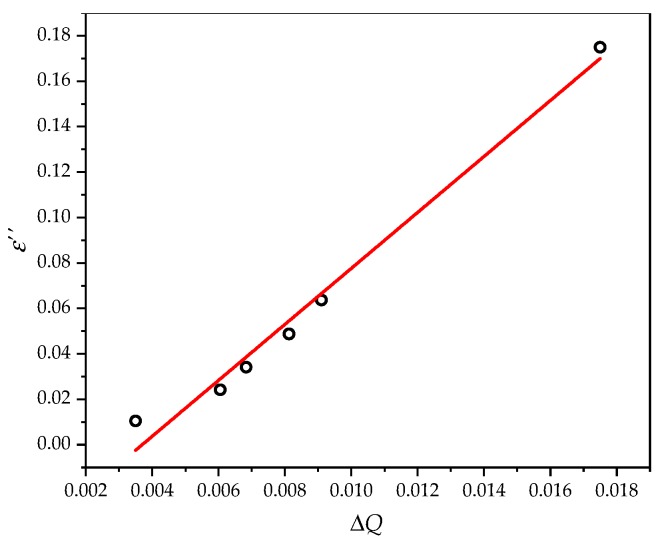
Linear fitting of the quality factor and virtual part of the relative dielectric constant.

Figure 12 is the group delay *τ*_S11_ under various dielectric constants and dielectric losses. Equation (13) is used to calculate the cavity quality factor *Q*. Fitting of the *ε*″–Δ*Q* relationship is shown in Figure 13. *ε*″ and Δ*Q* basically agree with the linear relationship of Equation (5). The fitting degree is 0.98444; the final form of Equation (5) is as follows:

(14) $$\varepsilon_{s}^{\prime \prime }=12.317\Delta Q-0.0455$$

**Figure 14:**
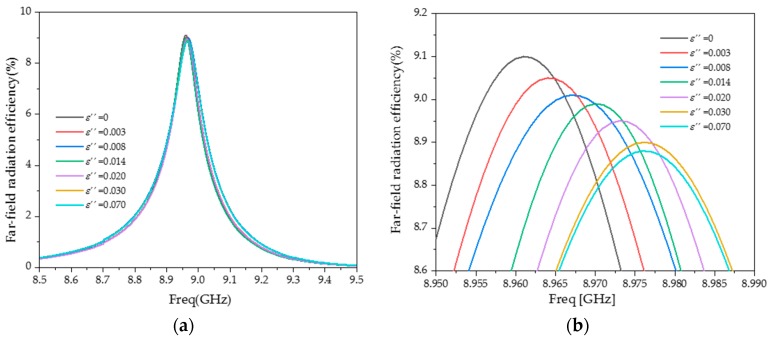
Simulation of far-field radiation efficiency under different *ε*″. (**a**) *f* = 8.5–9.5 GHz; (**b**) *f* = 8.95–8.99 GHz.

The simulation results of far-field radiation efficiency under different *ε*″ are shown in Figure 14. It can be seen that when *ε*″ changes from 0 to 0.07, the far-field radiation efficiency changes very little (0.23%). It shows that the radiation losses can be approximated as a constant, which does not affect the calculation of the *ε*″ of the MUT.

According to Equations (12) and (14), the calculation model of the relative dielectric constant under the circuit can be established. The MUT’s real part of the relative dielectric constant can be obtained by measuring the resonance frequency of S_11_, while the imaginary part can be obtained by measuring the quality factor.

## 4. Experiment and Discussion

**Figure 15:**
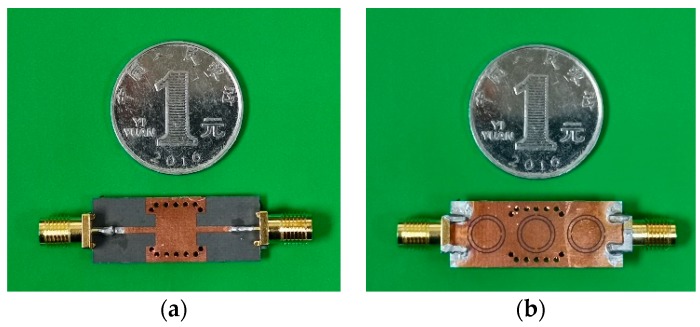
The fabricated sensor with multi-CSRR. (**a**) Top view; (**b**) bottom view.

**Figure 16:**
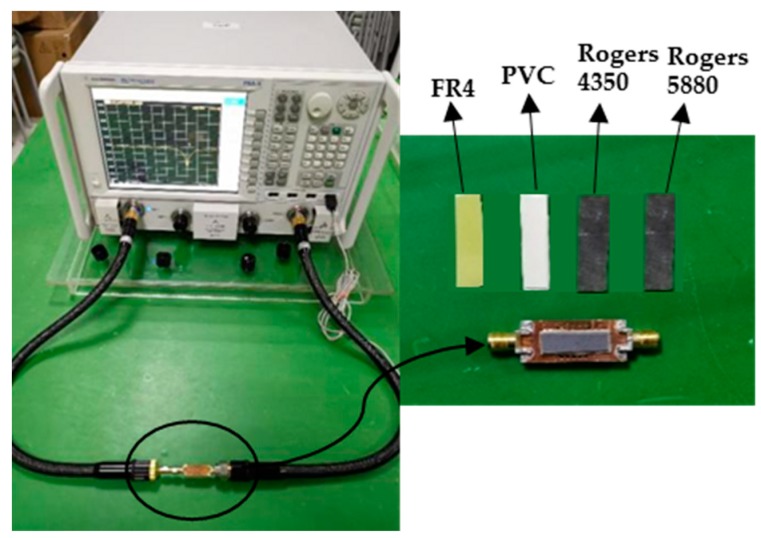
The MUT experimental validation with known standard permittivity for the fabricated sensor with multi-CSRR.

Figure 15 shows the fabricated sensor. The Agilent N5242A vector network analyzer is used for S parameter measurement, as shown in Figure 16.

**Figure 17:**
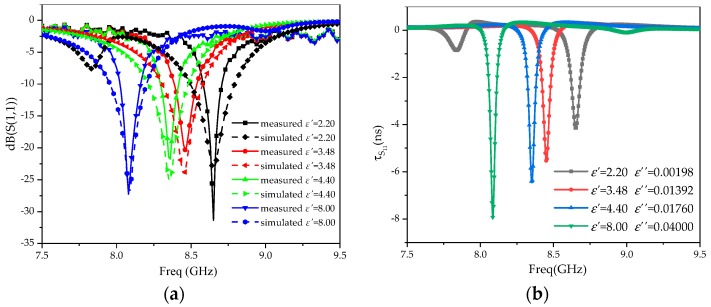
Measured results. (**a**) S_11_; (**b**) group delay (τ_S__11_).

A Rogers 5880 (complex dielectric constant 2.20–j0.00198), Rogers 4350 (complex dielectric constant 3.48–j0.01392), FR4 (complex dielectric constant 4.40–j0.0176), and PVC (complex dielectric constant 8.00–j0.04) were selected as the MUTs. The measured results of S_11_ and group delay are shown in Figure 17a,b, respectively, and the measured data are shown in Table 1.

As can be seen from Table 2, when the sensor is used to measure the dielectric constant of the MUT, the relative error of the real part is less than 2%, and the error value of the imaginary part is less than 0.00999, indicating a high accuracy. Compared to the measuring frequency range in related literature [11,12], a wider measuring range of the complex dielectric constant of the MUT is achieved. It makes the sensor more sensitive to the change in the dielectric constant by loading the CSRR on the metal ground. The SIW can achieve a more compact volume of the circuit structure. Of course, plate processing, SMA joint welding, size accuracy, data fitting of the MUT, etc., bring some errors to the measurement. The subject of the next important work will be how to further reduce the error of the permittivity imaginary part.

## 5. Conclusions

In this work, a sensor has been proposed to measure the complex dielectric constant of the unknown MUT with the resonant methods. A wider range of impedance matching and a deeper notch at the resonant frequency are achieved by loading three CSRR loops with different opening directions in the space of the SIW metal ground. The sensor achieved a wide range of high-sensitivity measurements by combining the resonant characteristics of the CSRR and the structural advantages of a SIW.

## Figures and Tables

**Table 1:** Extracted circuit parameters for CSRR.

Lumped Parameters	CSRR
*L* (pH)	12.86
*C* (PF)	60.99
*L_c_* (pH)	128.75
*C_c_* (PF)	2.66
*R* (Ω)	490.03

**Table 2:** Measured complex dielectric constant using fabricated sensor.

The MUT	*ε*′			*ε*″		
	Standard [12]	Measured	Relative Error	Standard	Measured	Error
Rogers 5880	2.20	2.19	0.45%	0.00198	0.0027896	0.00081
Rogers 4350	3.48	3.53	1.44%	0.01392	0.005156	0.00876
FR4	4.40	4.34	1.36%	0.0176	0.027454	0.00985
PVC	8.00	8.13	1.63%	0.04	0.049995	0.00999
